# The pregnane X receptor drives sexually dimorphic hepatic changes in lipid and xenobiotic metabolism in response to gut microbiota in mice

**DOI:** 10.1186/s40168-021-01050-9

**Published:** 2021-04-20

**Authors:** Sharon Ann Barretto, Frederic Lasserre, Marine Huillet, Marion Régnier, Arnaud Polizzi, Yannick Lippi, Anne Fougerat, Elodie Person, Sandrine Bruel, Colette Bétoulières, Claire Naylies, Céline Lukowicz, Sarra Smati, Laurence Guzylack, Maïwenn Olier, Vassilia Théodorou, Laila Mselli-Lakhal, Daniel Zalko, Walter Wahli, Nicolas Loiseau, Laurence Gamet-Payrastre, Hervé Guillou, Sandrine Ellero-Simatos

**Affiliations:** 1 Toxalim (Research Centre in Food Toxicology), INRAE, ENVT, INP-Purpan, UPS, Université de Toulouse, Toulouse, France; 2 Lee Kong Chian School of Medicine, Nanyang Technological University Singapore, Singapore, 308232 Singapore; 3 Center for Integrative Genomics, University of Lausanne, CH-1015 Lausanne, Switzerland

**Keywords:** Gut microbiota, Liver, Pregnane X receptor, NR1I2, Xenobiotic metabolism, Fatty acid metabolism, Transcriptomics

## Abstract

**Background:**

The gut microbiota–intestine–liver relationship is emerging as an important factor in multiple hepatic pathologies, but the hepatic sensors and effectors of microbial signals are not well defined.

**Results:**

By comparing publicly available liver transcriptomics data from conventional vs. germ-free mice, we identified pregnane X receptor (PXR, NR1I2) transcriptional activity as strongly affected by the absence of gut microbes. Microbiota depletion using antibiotics in *Pxr*^*+/+*^ *vs Pxr*^*-/-*^ C57BL/6J littermate mice followed by hepatic transcriptomics revealed that most microbiota-sensitive genes were PXR-dependent in the liver in males, but not in females. Pathway enrichment analysis suggested that microbiota–PXR interaction controlled fatty acid and xenobiotic metabolism. We confirmed that antibiotic treatment reduced liver triglyceride content and hampered xenobiotic metabolism in the liver from *Pxr*^+/+^ but not *Pxr*^-/-^ male mice.

**Conclusions:**

These findings identify PXR as a hepatic effector of microbiota-derived signals that regulate the host’s sexually dimorphic lipid and xenobiotic metabolisms in the liver. Thus, our results reveal a potential new mechanism for unexpected drug–drug or food–drug interactions.

Video abstract

**Supplementary Information:**

The online version contains supplementary material available at 10.1186/s40168-021-01050-9.

## Background

A vast ensemble of microorganisms, the gut microbiota, profoundly affects many aspects of its mammalian host physiology [1–4]. An imbalanced gut microbiota composition, together with host genetic characteristics and environmental factors (diet, drugs,…), is therefore suspected to lead to the development of a range of immune- and metabolic-mediated diseases. Among these, nonalcoholic fatty liver disease (NAFLD) is a common clinical syndrome currently affecting 25% of the general population [5] and characterized by the accumulation of triglycerides in hepatocytes. NAFLD contributes to insulin resistance, type 2 diabetes and cardiovascular risk and can evolve to nonalcoholic steatohepatitis (NASH) and eventually cirrhosis and hepatocellular carcinoma [6]. Several preclinical and human cross-sectional studies have demonstrated a relationship between alteration in the gut microbiota composition and NAFLD pathogenesis [7–10].

The liver receives 75% of its blood supply from the hepatic portal vein, which transports nutrients and xenobiotics from the gastrointestinal tract to the liver for processing before their metabolites reach the rest of the body. Multiple hepatic signaling pathways are sensitive to the gut microbiota, including bile acid synthesis [11], urea cycle [12], choline metabolism [13], oxidative stress [14], lipid metabolism [15] and xenobiotic metabolism [16, 17]. Several mechanisms are involved in the bidirectional relationship between the gut microbiota and the liver [18, 19]. Gut microbes can raise the permeability of the intestinal epithelium [20], which increases translocation of bacterial fragments and endotoxins into the portal circulation and liver: this metabolic endotoxemia can induce hepatic fat accumulation [21]. Translocation of gut microbiota-derived endotoxins can also lead to inflammasome deficiency and potentially the recruitment and activation of hepatic immune cells, which contribute to liver disease progression [22]. Another mechanism is through the production of bacterial metabolites derived from dietary substrates that can reach the portal blood and affect the host’s health [4]. The main end-products of bacterial metabolism are short-chain fatty acids, branched-chain amino acids, tryptophan-derived metabolites and choline derivatives, all of which have been associated with liver physiology and metabolic diseases [10, 13, 23–25].

Thus, the gut microbiota–intestine–liver relationship is emerging as an important factor in physiology but the molecular players involved in these interactions have yet to be identified. Transcription factors from the nuclear receptor superfamily are natural candidates, as they can sense fluctuating levels of nutrients and xenobiotics and promptly adapt hepatic metabolism by modulating gene transcription. Here, we took a global approach to identify potential hepatic effectors of gut microbial signals by performing transcription factor enrichment analysis on previously published mRNA expression datasets from germ-free (GF) vs. conventional (Conv.) mice. We identified PXR as a nuclear receptor whose transcriptional activity was strongly affected in the absence of gut microbes, in accordance with previous work that identified gut microbial metabolites activating PXR in the intestinal epithelium [26]. We used *Pxr*^+/+^ *vs. Pxr*^-/-^ C57BL/6J mice treated with antibiotics (ATB) to elucidate metabolic pathways controlled by the microbiota–PXR interactions and demonstrated that, in physiological conditions, this interaction affects both hepatic fatty acid and xenobiotic metabolism in a sexually dimorphic way.

## Methods

### *In vivo* studies

All mice were housed at 21–23 °C on a 12-h light (ZT0–ZT12)/ 12-h dark (ZT12–ZT24) cycle and allowed free access to the diet (Teklad Global 18% protein rodent diet) and tap water. ZT stands for Zeitgeber time; ZT0 is defined as the time when the lights are turned on.

For the experiment with pharmacological activation of PXR using pregnenolone 16α-carbonitrile (PCN), the control mice were 6-week-old wild-type C57BL/6J male mice purchased from Charles River and are named as wild-type “WT” mice throughout the manuscript. The *Pxr*^*-/-*^ animals (backcrossed on the C57BL/6J background) were originally engineered in Pr. Meyer’s laboratory [27]. Six-week-old WT male mice and *Pxr*^*--/-*^ animals were acclimatized for 2 weeks, then randomly allocated to the different experimental groups: WT CONT, WT PCN, *Pxr*^*--/-*^ CONT and *Pxr*^*--/-*^ PCN (*n* = 6 (one cage) per group). PCN-treated mice received a daily intraperitoneal injection of PCN (100 mg/kg, Bertin Bioreagent) in corn oil (Sigma Aldrich) for 4 days, while control mice received corn oil only. Mice were killed at ZT6, 6 h after the last PCN injection.

To avoid potential biases due to different breeding facilities, a specific breeding strategy was used for the rest of the experiments. The *Pxr*^*-/-*^ mice were crossed with C57BL/6J mice, thus generating new *Pxr*^*+/-*^ mice in our animal facility. These new *Pxr*^+/-^ mice were bred together and gave birth to true *Pxr*^+/+^ and *Pxr*^-/-^ littermate mice, which were then separated by sex and genotype as soon as possible after weaning and genotyping. Therefore, at 5 weeks, mice were randomly allocated to the different experimental groups: male *Pxr*^*+/+*^ control (Cont), male *Pxr*^*+/+*^ ATB, male *Pxr*^*-/-*^ Cont, male *Pxr*^*-/-*^ ATB, female *Pxr*^*+/+*^ Cont, female *Pxr*^*+/+*^ ATB, female *Pxr*^*-/-*^ Cont and female *Pxr*^*-/-*^ ATB (each experimental group containing *n* = 8 animals). Moreover, to avoid potential cage effects, each experimental group consisted of 2 separated cages. Six-month-old mice were subsequently treated or not with ATB in drinking water for 2 weeks as described previously [28]: three antibiotics were dissolved in tap water and were provided in the animal bottles for 2 weeks: ampicillin (1g/L, Euromedex), neomycin (1 g/L, Sigma-Aldrich) and vancomycin (0.5 g/L, MP Biomedicals). The antibiotic solutions were prepared fresh every 3 days. Antibiotics delivery was controlled through following of water intake per cage (Additional file 1A&C). Efficiency of antibiotic treatment was verified through measurement of caecal weight, fecal colony counting and caecal short-chain fatty acid determination using ^1^H-NMR as previously described [29] (Additional file 1). Mice were sacrificed at ZT6.

### Blood and tissue sampling

Blood was collected at the submandibular vein into lithium heparin-coated tubes (BD Microtainer, Franklin Lake, NJ, USA). Plasma was prepared by centrifugation (1500 *g*, 15 min, 4 °C) and stored at – 80 °C. Following euthanasia by cervical dislocation, liver tissue and ceacal content were weighed, snap-frozen in liquid nitrogen and stored at – 80 °C until used.

### Plasma analysis

Alanine transaminase (ALT), high- or low-density lipoprotein (HDL-LDL), total cholesterol, triglycerides and free fatty acids (FFA) were determined using a Pentra 400 biochemical analyzer (Anexplo facility, Toulouse, France).

### Liver neutral lipids

Hepatic samples were homogenized in 2:1 (v/v) methanol/ethylene glycol-bis(B-aminoethyl ether)-N,N,N’,N’-tetraacetic acid (EGTA; 5 mM), lipids corresponding to an equivalent of 2 mg of tissue were extracted (Bligh and Dryer,1959) in chloroform : methanol : water (2.5:2.5:2.1, v/v/v) in the presence of internal standards glyceryl trinonadecanoate, stigmasterol and cholesteryl heptadecanoate (Sigma). Triglycerides, free cholesterol and cholesterol esters were analyzed by gas chromatography on a Focus Thermo Electron system using a Zebron-1 Phenomenex (Phenomenex Zebron-1, England) fused-silica capillary column [5 m; 0:32 mm internal diameter (i.d.); 0:50 lm film thickness]. The oven temperature was programmed to rise from 200 to 350 °C at a rate of 5 °C/min and the carrier gas was hydrogen (0.5 bar). The injector and detector were at 315 °C and 345 °C, respectively.

### Liver fatty acid analysis

To measure all hepatic fatty acid methyl ester (FAME) molecular species, lipids that corresponded to an equivalent of 1 mg of liver were extracted in the presence of the internal standard glyceryl tri-heptadecanoate (2 μg). The lipid extract was transmethylated with 1 mL boron trifluoride (BF3) in methanol (14% solution; Sigma Aldrich) and 1 mL heptane for 60 min at 80 °C and evaporated to dryness. The FAMEs were extracted with heptane/water (2:1). The organic phase was evaporated to dryness and dissolved in 50 μL ethyl acetate. A sample (1 μL) of total FAME was analyzed with gas–liquid chromatography (Clarus 600 system (PerkinElmer), with FAMEWAX fused silica capillary columns (Restek), 30 m × 0:32 mm i.d., 0:25 lm film thickness). Oven temperature was programmed to increase from 110 to 220 °C at a rate of 2 °C/min and the carrier gas was hydrogen (7.25 pounds per square inch (psi)). Injector and detector temperatures were 225 °C and 245 °C, respectively.

### Gene expression

Total RNA was extracted using TRI Reagent® (Molecular Research Center, Cincinnati, OH, USA). RNAs were quantified using Nanodrop (NanoDropTM 1000; Thermo Scientific). Two micrograms of total RNA were reverse transcribed using the High-Capacity cDNA Reverse Transcription Kit (Applied Biosystems TM). The SYBR Green (Applied Biosystems, California) assay primers are presented in Additional file 2. Amplification was performed using an ABI Prism 7300 Real-Time PCR System (Applied Biosystems^TM^). Quantitative real-time polymerase chain reaction (qPCR) data were normalized to TATA-box-binding protein mRNA levels and analyzed with LinRegPCR (version 2015.3).

### Microarray

Microarray experiments were conducted on *n* = 6 mice per group (3 per cage). Total RNA was extracted with TRIzol reagent (Invitrogen, Carlsbad, CA, USA). Gene expression profiles were obtained at the GeT-TRiX facility (GénoToul, Génopole Toulouse Midi-Pyrénées, France) using Sureprint G3 Mouse GE v2 microarrays (8 × 60 K; design; 074,809; Agilent Technologies, Santa Clara, CA, USA) following the manufacturer instructions. Microarray data and experimental details are available in NCBI’s Gene Expression Omnibus (Edgar, Domrachev, & Lash, 2002) and are accessible through GEO Series accession numbers GSE123804 and GSE156837.

### DNA extraction from caecal content

DNA was isolated from the caecal content of mice using QiAamp Fast Stool Kit (Qiagen, Hilden, Germany) using manufacturer instructions after mechanical lysis with 5-mm steel beads for 3 min at 30 Hz with a Tissue Lyser (Qiagen). The quality and quantity of DNA extracts were analyzed by agarose gel electrophoresis (1% agarose in 0.5X TBE) and NanoDrop 2000 UV spectrophotometer (Thermo Scientific).

### 16S rDNA gene sequencing

The microbial population present in the caecal content of mice was determined using next-generation high-throughput sequencing of variable regions of the 16S rRNA bacterial gene for *n* = 7 mice per group. DNA from feces was isolated and amplified in a strictly controlled environment at Vaiomer SAS (Labège, France) using a stringent contamination-aware approach described and discussed previously [30]. The workflow performed at VAIOMER (France) included library construction and sequencing and PCR amplification performed using 16S universal primers targeting the V3–V4 region of the bacterial 16S ribosomal gene (Vaiomer universal 16S primers). The joint pair length was set to encompass a 476-pb amplicon (using *Escherichia coli* 16S as a reference) using a 2 × 300 paired-end MiSeq kit v3. For each sample, a sequencing library was generated by addition of sequencing adapters. The detection of the sequencing fragments was performed using MiSeq Illumina technology. The targeted metagenomic sequences from microbiota were analyzed using the bioinformatics pipeline established by Vaiomer from the FROGS v1.4.0 guidelines [31]. Briefly, after demultiplexing of the bar-coded Illumina paired reads, single-read sequences were cleaned and paired for each sample independently into longer fragments. Operational taxonomic units (OTU) were produced via single-linkage clustering and taxonomic assignment was performed in order to determine community profiles. The PhyloSeq v1.22.3 R package was used to provide a set of classes and tools to facilitate the import, storage, analysis and graphical display of microbiome census data (including alpha and beta diversity analysis). The samples with fewer than 5000 sequences after FROGS processing were not included in the statistics (rarefaction analysis, alpha diversity, beta diversity-multidimensional scaling). The raw sequencing data are available at EMBL-EBI’s European Nucleotide Archive (ENA) and are accessible through accession number PRJEB39629.

### Testosterone hydroxylation assay

#### Chemicals

Acetic acid, ethanol, NaCl, potassium dihydrogen phosphate, dibasic potassium phosphate, monobasic sodium phosphate, dibasic heptahydrate sodium phosphate, potassium sodium tartrate tetrahydrate, glycerol, Folin & Ciocalteu’s phenol reagent, albumin from bovine serum, anhydrous sodium carbonate, NADP, D-glucose 6-phosphate sodium salt, glucose-6-phosphate dehydrogenase, MgCl, 1-chloro-2,4-dinitrobenzene, reduced L-glutathione and testosterone were purchased from Sigma-Aldrich Merck (Saint Quentin Fallavier, France). Acetonitrile and methanol (high-performance liquid chromatography (HPLC) grade) were purchased from Thermo Fisher Scientific (Waltham, MA, USA). Ammonium acetate, sodium hydroxide and copper sulfate were purchased from VWR (Fontenay-sous-Bois, France). Flo-Scint™ II and Ultima Gold™ liquid scintillation cocktails were purchased from PerkinElmer (Courtabœuf, France). Ultrapure water produced by a Milli-Q system (Millipore, Saint-Quentin-en-Yvelines, France) was used for the preparation of HPLC mobile phases. [^14^C]-testosterone (specific activity: 2.18 GBq/mmol; 98.8% pure) was purchased from PerkinElmer. Standards of testosterone metabolites were purchased from Steraloids (Newport, RI, USA) or from Sigma-Aldrich.

#### Subcellular fraction preparation and protein content

Following mice euthanasia, livers were collected and immediately perfused using 0.9% NaCl. They were weighed and frozen in liquid nitrogen until subcellular fraction preparation. Livers were thawed and homogenized at 4 °C using a Potter-Elvehjem Teflon glass homogenizer in 4 volumes/g of ice-cold 0.1 M sodium/phosphate buffer, pH 7.4. Microsomal and cytosolic hepatic fractions were obtained after two centrifugation steps at 4 °C (20 min at 9000*g* and 70 min at 105,000*g*). Microsomes were resuspended with gentle homogenization in 1 mL/g of ice-cold 0.1 M sodium/phosphate buffer, pH 7.4 with 20% glycerol (v/v). Subcellular fractions were stored at – 80 °C until use. The protein content of subcellular fractions was determined using the Lowry et al. (1951) method [32].

#### [^14^C]-testosterone incubations

Mice hepatic microsomes (0.5 mg proteins/mL) were incubated at 37 °C under shaking with 10 μM [^14^C]-testosterone (3.58 kBq per incubation in 5 μL ethanol) fortified with unlabeled testosterone. Incubations were performed in a final volume of 0.5 mL in 0.1 M sodium/phosphate buffer pH 7.4 with 5 mM MgCl_2_. Incubations were initiated using a NADPH generating system: 1.3 mM NADP, 5 mM glucose-6-phosphate and 1 IU glucose-6-phosphate dehydrogenase. The kinetics of testosterone metabolite formation was established between 10 and 60 min. An incubation time of 20 min was selected in order to ensure a linear formation of testosterone metabolites. Incubations were stopped with 1.5 mL methanol, kept 30 min on ice and centrifuged 10 min at 6500*g*, 4 °C. For the liquid chromatography-mass spectroscopy (LC-MS) confirmation of major testosterone metabolite structures, additional incubations were carried out using unlabeled testosterone (50 μM) and pooled microsomes (1 mg proteins/mL, pool of 10 PXR control mice) for 30 min, in the same conditions.

#### Radio-HPLC profiling and quantification

Incubation media were individually analyzed by radio-HPLC for testosterone metabolite profiling and quantification. Reversed-phase high-performance liquid chromatography analyses were performed on an Ultimate-3000 system (Thermo Fisher Scientific) coupled with a flow scintillation analyzer Flo-One Radiomatic™ 610TR (PerkinElmer). The HPLC system consisted of a Nucleoshell RP18 column (250 × 4.6 mm, 5 μm, Macherey-Nagel, Hoerdt, France) coupled to a C18 guard precolumn (Nucleoshell RP18 5 μm EC 4/3, Macherey-Nagel), maintained at 35 °C. Mobile phases were (A) ammonium acetate buffer (20 mM, adjusted to pH 3.5 with acetic acid) and (B) acetonitrile. The flow rate was 1 mL/min and the injection volume was 500 μL. The gradient 0–35 min A:B from 80:20 to 60:40 (v/v); 35–45 min from 60:40 to 40:60; 45–48 min from 40:60 to 100% B. The system was returned to the initial condition at 51 min and held for another 4 min. Flo-Scint™ II (PerkinElmer) was used as scintillation cocktail, at a flow rate of 2 mL/min, and with a 500-μL detection cell. Each incubation medium (800 μL, *ca.* 1.5 kBq) was evaporated to dryness and reconstituted in mobile phase A:B 80:20 (v/v) prior to HPLC injection. Reversed-phase (RP)-HPLC profiles were processed with the A500 software (PerkinElmer). All peaks above 4% of the detected radioactivity were quantified for each animal.

#### Mass spectrometry analysis

Unlabeled testosterone incubation media were analyzed by LC-MS using the same HPLC conditions. Testosterone metabolite structures were established based on their mass and on the similarity of their retention time with authentic standards. Media were analyzed by LC-MS using a RSLC3000 HPLC system coupled to an HRMS system LTQ Orbitrap XL mass spectrometer (Thermo Fisher Scientific) with a post-column split (0.2 mL/min in the source) using positive electrospray ionization (ESI). The injection volume was 50 μL, the source voltage was 1.80 kV, the capillary voltage was 30V, the capillary temperature was 350 °C, the sheath gas (N_2_) flow (arbitrary unit) was 50, the auxiliary gas (N_2_) flow (arbitrary unit) was 40, the sweep gas (N_2_) flow (arbitrary unit) was 0, and the tube lens offset was 115 V.

### GST activity assay

Glutathione S-transferases’ (GST) specific activities were assessed in cytosolic fractions in 96-well plates. The assay is based on the GST-catalyzed reaction between GSH and the probe substrate CDNB (1-chloro-2,4-dinitrobenzene), which is metabolized by a broad range of GST isozymes. Protein content was between 0.6 and 1.6 μg protein per incubation (controls: 0), to allow a linear measurement of the formation of CDNB-GSH. Incubations were performed in 0.1 M potassium phosphate buffer, pH 6.5, using 2.5 mM reduced L-glutathione. The reaction was initiated by the addition of ice-cold CDNB (2.5 μM final). CDNB-GSH formation was measured by the change in absorbance at 340 nm, recorded every min over 10 min, using a Tecan Infinite 200. Mean GST specific activities (*n* = 5 mice per group) were expressed in nanomoles (nmol) of product formed per minute per milligram of proteins (nmol/min/mg).

### Functional and gene set enrichment analysis

Gene expression datasets comparing hepatic signatures of GF vs. Conv. animals were found on the Gene Expression Omnibus data repository accessed in September 2019. Only data obtained in wild-type mice were considered. Data obtained at different ZT were pooled to increase sample size. Five datasets were available and used in the present analysis (GSE31115, GSE5390, GSE71628 and GSE114400 (males and females)). Differentially expressed genes were calculated using the GEO2R tool for microarray data and using BioJupies [33] for RNA sequencing data. Genes with adjusted *p* values < 0.05 and Ifold changeI > 1.2 were considered differentially expressed. Analyses of transcription factor enrichment were performed with Enrichr [34], by interrogating the TRRUST Transcription Factors 2019 database and reporting adjusted *p* values for mouse transcription factors while discarding human transcription factors.

### Microarray data analyses

Microarray data were processed using R (http://www.r-project.org) and Bioconductor packages (http://www.bioconductor.org, v3.0). Raw data (median signal intensity) were filtered, log2 transformed, corrected for batch effects (microarray washing bath) and normalized using the CrossNorm method [35]. The linear model was fitted using the limma lmFit function [36]. Pair-wise comparisons between biological conditions were applied using specific contrasts. A correction for multiple testing was applied using the Benjamini–Hochberg procedure for false discovery rate (FDR). Probes with FDR ≤ 0.05 and |fold-change| > 1.2 were considered to be differentially expressed between conditions. The gene set list of sexually dimorphic liver genes is based on the comparison between *Pxr*^+/+^ Cont males and *Pxr*^+/+^ Cont females. Genes that show significantly different expression patterns (adjusted *p* value < 0.05 and Ifold-changeI > 1.2) between males and females were considered as male- and female-biased, respectively. The full list of probes with annotated genes, and fold changes, raw *p* values and corrected *p* values for all group comparisons are provided in Additional file 3.

### Sequencing data analyses

The operational taxonomic unit (OTU) files were formatted for linear discriminant analysis effect size (LEfSe) analysis using the per-sample normalization of sum values option, using the R package Phyloseq v1.22.3. The linear discriminant analysis (LDA) effect size (LEfSe) was run using default values (alpha value of 0.05 for both the factorial Kruskal-Wallis test among classes and the pairwise Wilcoxon-Mann-Whitney test between subclasses, threshold of 2.0 for the logarithmic LDA score for discriminative features) and the strategy for multi-class analysis was set to ‘all-against-all’. LEfSe cladograms from the LDA effect size data were generated with Bacteria as the tree root.

### Multi-omics analyses

Bidirectional correlations between caecal OTUs and hepatic transcriptome were investigated using N-integration discriminant analysis with DIABLO, an algorithm that aims to identify a highly correlated multi-omics signature discriminating several experimental groups [37] using the R package Mixomics v6.10.9 [38]. OTUs with a minimal presence of 1% in all samples were kept. Samples were excluded from the microbiota-data if they were not measured in the microarray analyses (i.e. integration was performed on *n* = 6 samples/group). DIABLO models were fitted in males and females separately; we used 2 components in the models, and for the estimation of model parameters, the cross-validation procedure (CV) method was used. For the Circos plot and correlation networks, only correlations with a Spearman’s rank correlation coefficient > 0.9 were plotted.

### Other statistical analyses

GraphPad Prism version 7.0 (San Diego, CA, USA) was used for all remaining analyses and preparation of graphs. For all data displayed in graphs, results are expressed as the mean ± s.e.m. The Kolmogorov–Smirnov test of normality was applied to all data sets, and in cases where the data did not demonstrate a normal distribution, nonparametric tests were used to analyze statistical differences. One-way analysis of variance (ANOVA) and the *post hoc* Tukey test or nonparametric Kruskal–Wallis test followed by a *post hoc* Dunn’s test was used. Differences corresponding to *p* < 0.05 were considered significant.

## Results

### Absence of gut microbiota alters PXR-dependent gene expression in the mouse liver

**Fig. 1:**
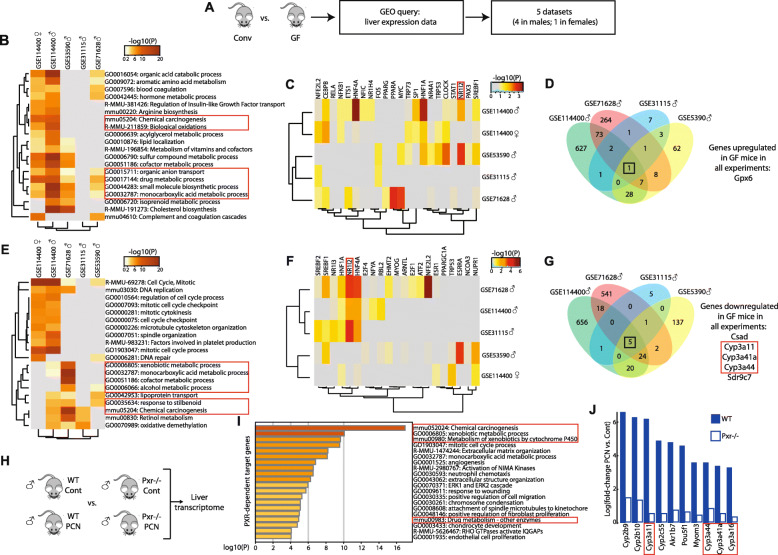
Absence of gut microbiota significantly alters PXR target-gene expression in the liver. **a** Five publicly available gene expression datasets comparing the livers of germ-free (GF) vs*.* conventional (Conv.) mice were selected. **b** Pathway enrichment analysis and **c** transcription-factor enrichment analysis performed on the genes overexpressed in GF vs. Conv mice. **d** Venn diagram showing the number of genes overexpressed in GF *vs*. Conv male mice. **e** Pathway enrichment analysis and **f** transcription factor enrichment analysis performed on the genes underexpressed in GF vs. Conv. mice. **g** Venn diagram showing the number of genes underexpressed in GF *vs*. Conv male mice. **h–j** Confirmation of transcriptomic signature of PXR activation was obtained by comparing the hepatic transcriptome of WT vs. *Pxr*^-/-^ mice treated with PCN, the pharmacological agonist of PXR. **i** Pathway enrichment analysis on the genes overexpressed in PCN-treated vs. vehicle WT mice, but not in PCN-treated vs. vehicle *Pxr*^-/-^ mice. **j** Hepatic genes with the most significant fold change upon PCN treatment in WT mice. Pathways, transcription factors and genes related to drug and xenobiotic processing are framed in red

To investigate the biological functions impacted by gut microbial signals in the liver, as well as transcription factors that could be potential sensors of these signals, we first interrogated previously published microarray and RNAseq datasets comparing the hepatic transcriptomes of GF vs. Conv. mice (Fig. 1a–g). Process and pathway enrichment analysis using genes significantly up- and downregulated in GF mice (Fig. 1b and e, respectively) highlighted xenobiotic metabolism as significantly affected by the absence of gut microbes in most studies. Only one study used both male and female GF mice (GSE114400). Interestingly, in this dataset, gene ontology (GO) terms related to xenobiotic metabolism were significantly enriched in the list of downregulated genes when comparing male GF vs. Conv. males but not when GF vs. Conv. females were compared (Fig. 1e). Looking for upstream regulators of these genes, we found several transcription factors whose biological activity was significantly perturbed in GF mice, among which was PXR (official name NR1I2 [39]), a master regulator of hepatic xenobiotic metabolism (Fig. 1c and f). Again, results from the GSE114400 study illustrate sexually dimorphic results since PXR (NR1I2) was predicted as a significant upstream regulator of the genes that are both over- and under-expressed in GF compared with Conv. male mice (*p* = 0.01 for both up- and downregulated genes), but not in females (*p* > 0.05) (Fig. 1c and f). Accordingly, in all studies in males, the expression of cytochrome P450 (CYP) family 3 mRNA was decreased (Fig. 1g). This result is consistent with reduced PXR activity in GF mice, since PXR is a well-known regulator of CYP3 expression [40, 41], as confirmed here using WT *vs*. *Pxr*^-/-^ male mice treated with the pharmacological agonist of PXR (Fig. 1h–j). These results also suggested a possible sexual dimorphism in the gut microbiota–liver interactions, possibly involving PXR.

### PXR deletion induces sexually dimorphic changes in gut microbiota composition and the hepatic transcriptome

**Fig. 2:**
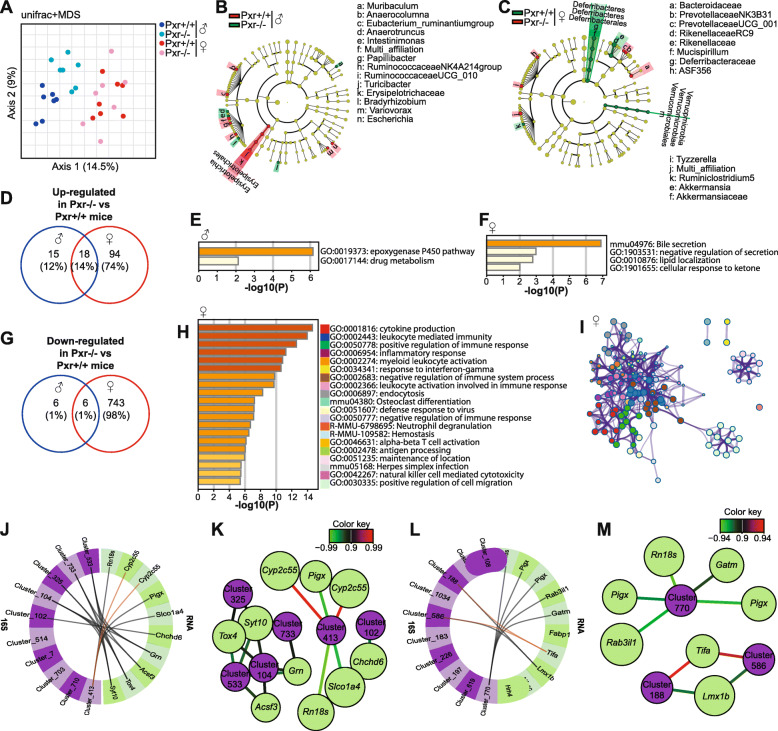
PXR deletion induces sexually dimorphic changes in gut microbiota composition and the hepatic transcriptome. a Unweighted UniFrac multidimensional scaling (MDS) plot based on OTUs (β-diversity). **b** and **c** Circular cladogram generated from LEfSe analysis showing the most differentially abundant genera significantly enriched in microbiota from *Pxr*^+/+^ (green) or *Pxr*^-/-^ (red) males (**b**) or females (**c**). Corresponding LDA scores are presented in Additional file 4F&G. **d** Venn diagram representing the number of genes overexpressed in the liver from *Pxr*^-/-^ vs. *Pxr*^+/+^ mice. **e** Pathway enrichment analysis of the 15 genes overexpressed in *Pxr*^-/-^ *vs*. *Pxr*^+/+^ males. **f** Pathway enrichment analysis of the 94 genes overexpressed in *Pxr*^-/-^ *vs*. *Pxr*^+/+^ females. **g** Venn diagram representing the number of genes underexpressed in the liver from *Pxr*^-/-^ *vs*. *Pxr*^+/+^ mice. **h** Pathway enrichment analysis of the 743 genes overexpressed in *Pxr*^-/-^ *vs*. *Pxr*^+/+^ females. **i** Network plot of the enriched terms depicted in (**h**) colored by cluster ID. **j** and **l** Circos plot representing the correlations between caecal bacterial OTUs (blue side quadrant) and hepatic mRNA (green side quadrant) variables in males (**j**) and females (**l**). Positive and negative correlations are illustrated with orange and black lines, resp. **k** and **m** Relevance network of bacterial OTUs and hepatic transcripts in males (**k**) and females (**m**)

To further investigate the PXR–gut microbiota relationship, we used C57BL/6J *Pxr*^+/+^ *vs. Pxr*^-/-^ littermates derived from *Pxr*^*+/-*^ dams and separated from their mothers and from their littermate siblings by sex and genotype after weaning, and analyzed their caecal content microbiota at 6 months. PXR deletion did not change bacterial richness, nor evenness (α-diversity, Additional file 4A&B). Analyses of the β-diversity revealed that the caecal microbiota clustered separately according to sex on the first axis and that separation between the 2 genotypes was observed for males on the second axis (Fig. 2a). Additional PLS-DA analysis confirmed that discrimination between *Pxr*^+/+^ and *Pxr*^-/-^ females was observed on the second PLS-DA axis, while clustering of *Pxr*^*+/+*^ *vs. Pxr*^*-/-*^ males was seen on the third axis (Additional file 4C). Changes in proportion of taxa were characterized using the LEfSe algorithm (Fig. 2b and c and Additional file 4F&G) or Mann–Whitney test (Additional file 4D-H). The major phyla were unaffected upon PXR deletion in males (*p* > 0.05, Additional file 4D), but many taxa were different in abundance using the LEfSe algorithm (*α* < 0.05, Fig. 2b and *p* < 0.05, Additional file 4F) and the Mann–Whitney test (*p* < 0.05, Additional file 4H). Interestingly, these taxa were unaffected by PXR deletion in females (p>0.05, Additional file 4H). At the phylum level, *Pxr*^-/-^ females displayed higher relative levels of *Deferribacteres* (*p* = 0.003) and *Verrucomicrobia* (*p* = 0.006) and several changes at the OTU level compared with *Pxr*^+/+^ females (*p* < 0.05, Additional file 4E). Thus, PXR deletion induced sex-specific changes in the gut microbiota composition.

PXR deletion also impacted the liver transcriptome in a sexually dimorphic manner (Fig. 2d–i), much more in female than in male mice (Figs. 2d–f). In *Pxr*^*-/-*^ males, 53 genes had significantly higher mRNA levels compared to *Pxr*^*+/+*^ males (Fig. 2d, Additional file 5A). These genes were involved in linoleic acid metabolism (*p* = 1.2 × 10^-11^) and xenobiotic metabolism (*p* = 3.2 × 10^-7^) (Fig. 2e, Additional file 5B). *Pxr*^*-/-*^ females displayed 110 genes with higher expression compared with *Pxr*^*+/+*^ females (Additional file 5C). These genes were involved in various metabolic pathways related to bile acid and xenobiotic metabolisms (“chemical carcinogenesis”, *p* = 1.4 × 10^-8^; “xenobiotics”, *p* = 1.6 × 10^-7^, “bile secretion”, *p* = 1.1 × 10^-6^), as well as “lipid localization” and “catabolism” (*p* = 6.5 × 10^-4^ and *p* = 4.6 × 10^-3^) (Fig. 2f, Additional file 5D). Surprisingly, PXR deletion induced a drastic downregulation of gene expression, almost exclusively in female mice (Fig. 2g, Additional file 5G). These female-specific regulated genes were involved in inflammation- and immunity-related pathways (Fig. 2h, Additional file 5G&H), with the following GO terms as the most significantly enriched: cytokine production (*p* = 1.9 × 10^-12^), leukocyte-mediated immunity (*p* = 3 ×10^-15^) and positive regulation of immune response (*p* = 1.5 × 10^-13^). These different pathways were highly interconnected (Fig. 2i). Our data therefore reveal a strong, female-specific constitutive role of PXR in maintaining immune gene expression.

The liver is a sexually dimorphic organ [42]. To further analyze the impact of PXR deletion on sexually dimorphic gene expression, we first defined hepatic female- and male-biased genes (see Methods section and Additional file 6). We found 784 male-specific genes in *Pxr*^*+/+*^ mice (Additional file 6A), a vast proportion of which (68%) remained male-specific in *Pxr*^*-/-*^ mice (Additional file 6 B, Additional file 7A). By contrast, only 47% of the 3691 genes defined as female-specific were still found as female-specific in *Pxr*^*-/-*^ animals (Additional file 6D&E, Additional file 7B). Hierarchical clustering of all genes significantly regulated in both male and female *Pxr*^*+/+*^ and *Pxr*^*-/-*^ animals confirmed that, although the vast majority of the sexually dimorphic genes remained sexually dimorphic upon PXR deletion (clusters 2, 3 and 5), a large cluster of 1419 genes (cluster 1) lost its female-biased expression, due to a significantly lower expression in *Pxr*^*-/-*^ compared with *Pxr*^*+/+*^ females, while no difference was observed in males (Additional file 7C&D). Interestingly, these genes are mostly those involved in immune processes, as described previously (Additional file 7E). Therefore, PXR deletion led to partial masculinization of the female liver for a portion of genes involved in hepatic immunity.

Bidirectional correlations between bacterial taxa and hepatic mRNA were also sex-specific (Fig. 2j–m). In males, there was a strong positive correlation between bacteria from the OTU “Cluster 413” (*Ruminococcaceae UCG-005*) and the mRNA level of Cyp2c55, a well-described PXR target gene (*r*^2^ = 0.98, Fig. 1j and k). Altogether, our data show a bidirectional relationship between gut microbes and hepatic PXR that mutually influence each other in a sexually dimorphic way.

### PXR deletion affects the hepatic transcriptional response to gut microbiota depletion

**Fig. 3:**
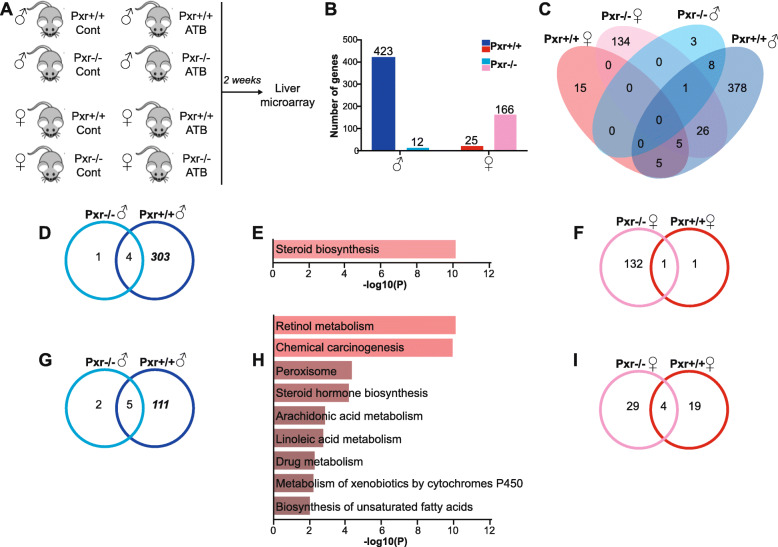
Sexually dimorphic changes in hepatic gene expression associated with microbiota depletion depends on PXR. a Experimental design. *ATB* antibiotics. **b** Number of genes regulated in ATB-treated vs. control mice. **c** Venn diagram representing the number of genes regulated in ATB vs. control mice. **d** Venn diagram representing the number of genes overexpressed in ATB *vs*. control males. **e** Pathway enrichment analysis of the 303 hepatic genes overexpressed in ATB-treated Pxr^+/+^ males. **f** Venn diagram representing the number of genes overexpressed in ATB *vs*. control females. **g** Venn diagram representing the number of genes underexpressed in ATB *vs*. control males. **h** Pathway enrichment analysis of the 111 hepatic genes underexpressed in ATB-treated *Pxr*^+/+^ males. **i** Venn diagram representing the number of genes underexpressed in ATB vs. control females

We then depleted the gut microbiota by antibiotic (ATB) treatment in *Pxr*^+/+^ and *Pxr*^-/-^ mice. As expected, antibiotic treatment significantly increased caecal weight, decreased fecal colony counts and completely abolished short-chain fatty acids in caecal contents, therefore demonstrating successful and homogenous depletion of gut microbes in all animals (Additional file 1). We characterized the extent and impact of gut microbiota sensing by PXR on the host’s hepatic transcriptome. Microbiota depletion affected a much higher number of genes in *Pxr*^+/+^ males than in *Pxr*^*+/+*^ females (Fig. 3b and c, Additional file 8). We confirmed this sex-biased interaction by reanalyzing publicly available data that compared mRNA expression in GF vs*.* Conv. male and female mice [43]. The number of significantly differentially expressed genes between GF and Conv. males was indeed almost twice that between GF and Conv. females (Additional file 9).

We next investigated the impact of microbiota depletion on sexually dimorphic gene expression, using the previously defined list of hepatic female- and male-biased genes (Additional file 6). A vast proportion of male-specific genes (70%) remained male-specific in ATB-treated *Pxr*^*+/+*^ mice (Additional file 10A). By contrast, only 49% of female-specific genes remained female-biased in ATB treated mice (Additional file 10B). Hierarchical clustering further revealed two clusters of female-specific genes that displayed increased expression upon ATB treatment in male mice only (cluster 4) or mostly (cluster 5), thereby mitigating sexually dimorphic expression of these genes and explaining the observed loss in female-specific genes in ATB-treated mice (Additional file 10C&D).

Surprisingly, in *Pxr*^-/-^ males, ATB treatment affected a very low number of genes compared with *Pxr*^+/+^ males (12 vs. 423 resp., Fig. 3b–d and g, Additional file 8). These PXR-dependent genes were mainly involved in lipid and xenobiotic metabolism. “Steroid biosynthesis” pathway was significantly enriched among the genes upregulated in ATB-treated males (*p* = 7.5 × 10-^6^, Fig. 3e; Additional file 8B), while chemical carcinogenesis (*p* = 6.4 × 10^-12^), retinol metabolism (*p* = 1.1 × 10^-10^), steroid hormone biosynthesis (*p* = 1.2 × 10^-5^) and metabolism of xenobiotics (*p* = 3.9 × 10^-5^) were among the top-regulated pathways from the genes downregulated in ATB-treated males (Additional file 8D, Fig. 3h). On the contrary, in *Pxr*^-/-^ females, ATB treatment affected more genes compared with *Pxr*^+/+^ females (166 vs. 25 resp., Fig. 3b, c, f and i, Additional file 8). These 166 genes comprised 57 sex-biased genes, which did not represent a significant enrichment in sex-biased genes (*p* = 0.99 using Fisher exact test). Moreover, pathway enrichment analysis failed to reveal significant PXR-dependent biological pathways affected upon microbiota depletion in females (Fig. 3f and i, Additional file 8G-K).

Since it has been previously reported that PXR is regulated by circadian rhythm in a sex-dependent manner [44], we confirmed that the expression of *Pxr* and 2 of its main target genes, *Cyp3a11* and *Cyp2c55,* was not significantly different between males and females at the time of sacrifice (Additional file 11). Thus, differences in PXR expression or activity are not likely to contribute to the observed sexually dimorphic microbiota- and PXR-dependent responses.

### PXR is involved in gut microbiota-dependent hepatic fatty acid metabolism

**Fig. 4:**
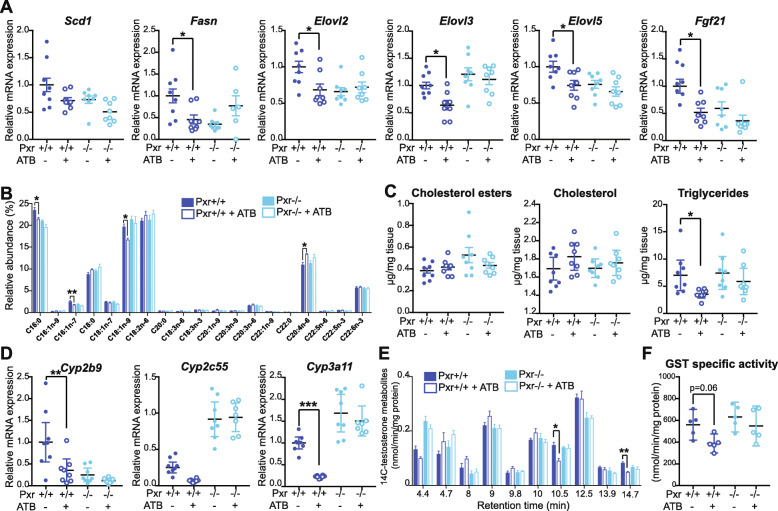
Gut microbiota-dependent changes in hepatic lipid and xenobiotic metabolism are controlled by PXR in male mice. **a** Hepatic gene expression assessed by qPCR. **b** Relative abundance of hepatic fatty acids. **c** Hepatic neutral lipid quantification. **d** Hepatic gene expression assessed by qPCR. **e [**^14^C]-testosterone hydroxylated metabolites obtained from incubation of ^14^C-testosterone with hepatic microsomes (*n* = 5 per group). **f** Glutathione S-transferase-specific activities assessed in hepatic cytosolic fractions (*n* = 5 per group)

Our data suggest that PXR sensing of the gut microbiota might be involved in fatty acid metabolism. ATB treatment or PXR deletion did not significantly impact circulating lipid levels (Additional file 2I&J). However, ATB-treated *Pxr*^+/+^, but not *Pxr*^-/-^, males, had decreased mRNA levels for several hepatic genes involved in fatty acid synthesis (*Fasn*) and elongation (*Elovl2, 3* and *5*) compared with the respective control males (Fig. 4a). Accordingly, we observed a lower relative abundance of three of the most abundant hepatic fatty acids (C16:0, *p*_adj_ = 7 × 10^-3^, C16:1n-7, *p*_adj_ = 0.02 and C18:1n-9, *p*_adj_ = 1.2 × 10^-4^) (Fig. 4b) and lower hepatic triglyceride content (*p* = 0.03, Fig. 4c) in ATB-treated *Pxr*^+/+^ males compared with *Pxr*^+/+^ control males, while these levels were similar between ATB-treated and control *Pxr*^-/-^ males. In females, there was no PXR-dependent difference in the expression of fatty acid-related genes nor in hepatic triglyceride levels (Additional file 12). Overall, gut microbiota depletion induced a significant remodeling of the hepatic lipids in a PXR-dependent manner in males.

### The gut microbiota–PXR interaction controls the host’s hepatic xenobiotic metabolism

Another interesting finding from our microarray data was the strong enrichment of genes related to xenobiotic metabolism. ATB-treated *Pxr*^+/+^, but not *Pxr*^-/-^, males displayed lower hepatic expression of genes involved in phase I xenobiotic metabolism (Fig. 4d). In order to test the functional consequences of the downregulation of these CYP genes, the regio-selective oxidative metabolism of [^14^C] testosterone in males was measured, using hepatic microsomal subcellular fractions. Testosterone metabolites with peaks at retention times of 10.5 and 14.7 min were significantly lower in ATB-treated vs. control *Pxr*^+/+^ males (*p*_adj_ = 1.5 × 10^-3^ and *p*_adj_ = 0.014 resp., Fig. 4e). The 10.5 min peak was formally identified as 6*β*-hydroxytestosterone (Additional file 13), a product of 6*β-*hydroxylase activity performed primarily by CYP3A11 in mice [16]. Finally, we observed a PXR-dependent decrease in glutathione transferase activity upon ATB treatment in male mice (*p*_adj_ = 0.06, Fig. 4f).

## Discussion

Early in development, the liver directly buds from the foregut. Once differentiated, these two organs are strongly interdependent and multiple lines of evidence demonstrate that disturbance of the gut–liver axis is involved in a number of metabolic diseases linked to obesity, including NAFLD [19]. In the liver, transcription factors from the nuclear receptor superfamily can sense fluctuating levels of nutrients and xenobiotics delivered via the portal vein and promptly adapt hepatic metabolism by modulating gene expression [45]. Therefore, some of these nuclear receptors might be crucial intermediates of microbial signals. Our work links the nuclear receptor PXR and the gut microbiota in a sexual dimorphic manner and highlights previously ignored aspects of the gut–liver cross-talk that could have major implications in sex-based medicine.

Our transcriptomic analysis indeed first revealed a strongly sexually dimorphic hepatic impact of PXR deletion. PXR is highly expressed in the liver and intestine of mammals [46] and was first characterized as a xenosensor that regulates the expression of xenobiotic-metabolizing enzymes and transporters, thereby facilitating the elimination of xenobiotics and endogenous toxic chemicals such as bile acids [40]. PXR activation has been shown to impact both xenobiotic and glucose and lipid metabolism in a sexually dimorphic way [44, 47, 48]. Here, we show that PXR has a broad, female-specific repressive action on hepatic genes involved in immunity. Interestingly, in mice deleted for PPARα, a similar effect was observed and attributed to SUMOylation of this receptor in female liver [49]. PXR is modified by multiple post-translational modifications, including SUMOylation [50], but whether these modifications are sex-specific *in vivo* is unknown to date. Moreover, our analysis also demonstrates that PXR deletion altered sexually dimorphic gene expression, leading to partial masculinization of the hepatic gene patterns in female mice. This corroborates previous findings [43]. Liver disease shows marked sex differences, male mice and humans being more sensitive to NAFLD, NASH, fibrosis [51, 52], hepatocellular carcinoma [53] and chemical-induced hepatic carcinogenesis [54]. Underlying these sex-biased phenotypic differences are hundreds of sexually dimorphic hepatic genes. There is a vast body of literature illustrating the protective role of estrogen in the liver [55], and females are thought to have a weaker hepatic inflammatory response, explaining, at least in part, why they are less susceptible to the progression of liver diseases. PXR, is a key player in inflammation [56, 57]. However, it remains to be determined whether female mice with deleted PXR are more prone to immune-driven liver diseases. Our current data suggest that, similar to PPARα [49], the repressive role for PXR in the female liver might have been overlooked.

Another interesting finding of our transcriptome analysis was the highly sexually dimorphic impact of gut microbiota depletion. Only 5–10% of the hepatic genes were regulated in common in males and females upon ATB treatment. Our results were further confirmed by re-analyzing prior data from GF mice [43]. Moreover, we have shown that microbiota depletion led to altered sexually dimorphic gene expression of the liver, mainly due to a cluster of genes that displayed increased expression upon ATB treatment in male mice only. Although the number of genes affected by this feminized expression pattern remained limited in the course of the 2-week ATB treatment used in our study, this confirmed previously published results in which GF males under chow or high-fat diet were shown to display extensive feminized hepatic gene expression compared with ConvR male mice [43]. The gut microbiota is therefore critical for maintaining sex-biased hepatic gene expression and these findings highlighted a potential role for PXR in the interplay between microbiota and sex-biased signaling pathways. Most studies on gene expression in the gut microbe–liver dialogue have used male animals [23, 43, 58, 59] and observed that xenobiotic metabolism, steroid biosynthesis and lipid metabolism were the most downregulated pathways in the livers of GF mice. Our current findings therefore raise the question, “How much of the current knowledge about gut microbial impact on the host’s liver and metabolism holds true in females?” Interestingly, a sex-specific association between gut microbes and fat distribution has recently been observed in humans [60].

We found that, in the absence of PXR, female livers displayed an enhanced response to the absence of gut microbiota compared with *Pxr*^*+/+*^ females. The reason for this is unclear since no obvious biological pathway or upstream regulator of these new microbiota-sensitive genes emerged from our analysis. Also, these genes were not enriched for sexually dimorphic genes. However, activation of PXR has been shown to interfere with sex hormone homeostasis [61], through regulation of CYPs and sulfotransferases (SULTs) important for the metabolic deactivation of androgens [62] and estrogens [63]. Therefore, we can hypothesize that PXR deletion also influenced steroid hormone balance and sexual maturation and, consequently, sexual dimorphism in gene expression. This complex gut microbiota–PXR–sex hormone homeostasis remains to be investigated. On the contrary, in the absence of PXR, the hepatic transcriptional response to microbiota depletion was almost completely abolished in male mice. By comparison, less than 5% of the colonic gene expression changes induced by the absence of gut microbiota required MyD88 signaling, a major adaptor in Toll-like receptor signaling [64]. Our result thus demonstrates that PXR is, at least quantitatively, a major effector of gut microbial signals in the male mouse liver. Since PXR is also highly expressed in the intestine and is known to increase gut permeability upon microbial ligand binding [26], it would be interesting to compare the proportion of microbially regulated PXR-controlled genes in the liver after hepatic-specific deletion of PXR.

Our pathway enrichment analysis of the microbially driven PXR-dependent hepatic function highlighted lipid metabolism. The gut microbiota promotes hepatic lipid accumulation in mice by controlling fatty acid desaturation [23, 65] and elongation [15]. Acetate originating from the microbial degradation of dietary fibres serves as a precursor for the hepatic synthesis of C16 and C18 fatty acids [15]. In our experiment, short-term ATB treatment was sufficient to reduce the expression of hepatic fatty acid elongases, hepatic triglyceride content and relative abundance of C16 fatty acid in males. Interestingly, our study highlights a potential role for PXR in these processes.

Finally, in GF and ATB-treated male animals, xenobiotic metabolism was strongly affected by the gut microbiota, in accord with previous studies in which Cyp3a11 is one of the most downregulated genes and proteins in GF male mice [16, 17, 23, 43, 66]. Here, we have demonstrated that PXR is the key mediator of these changes. In recent years, the gut microbial metabolism has been shown to influence the efficacy and toxicity of orally administered drugs [67, 68] and food pollutants [69]. However, our findings (this study [16, 17];) and others’ [66, 70] have shed light on another potential mechanism underlying food–drug or drug–drug interactions. In humans, CYP3A4, PXR’s prototypical target gene, metabolizes the vast majority of clinically administered drugs [71], and induction or repression of its activity is considered the main risk factor for drug–drug interaction [72, 73]. Our results indicate that gut microbes, via PXR, may partly control individuals’ metabolism of drugs and xenobiotics. It remains to be determined whether this has clinical relevance, in regard to either medications with narrow therapeutic indexes or potentially life-threatening toxicities, or to food contaminants found to perturb the composition of the gut microbiota and its metabolism, with toxicological consequences for the host [74, 75].

Recent studies on the mechanisms that link the body’s microbial communities to metabolism point to microbiota-derived metabolites as key players. The 3 currently most studied classes of metabolites involved in microbiota–host crosstalk are (i) short-chain fatty acids, produced by the microbial fermentation of dietary fibres, (ii) tryptophan metabolites generated by the gut microbes and (iii) bile acids, produced in the liver and transformed by the microbiota; (i) little is known about the interactions between short-chain fatty acids and PXR. However, butyrate induces PXR expression upon differentiation of CaCo-2 intestinal epithelial cells [76]. (ii) Gut microbes can convert tryptophan to indole derivatives, such as indole-3-propionic acid (IPA), which activates both the mouse and human PXR *in vitro* and decreases intestinal permeability in a PXR-dependent way *in vivo* [26]. In distant organs, IPA has been shown to regulate endothelium-dependent vasodilation *in vivo* [77]. Indoles, and IPA in particular, might be microbial ligands regulating PXR. However, it is not clear they could reach the liver at a sufficient concentration to activate the receptor. (iii) Bile acids interact with PXR directly [27, 78] or indirectly via regulation of the farnesoid X receptor [79]. Conversely, PXR induces genes involved in bile acid synthesis [79, 80], conjugation and transport thereby enhancing their elimination [27, 81]. Therefore, bile acids could play a role in microbiota–PXR crosstalk. In-depth metabolomic profiling of small molecules circulating in the portal veins of ATB-treated and control mice, followed by *in vitro* screening of differential metabolites using reporter cell lines, could identify microbial signals that activate hepatic PXR.

## Conclusion

In conclusion, we have first identified PXR as a potential repressor of hepatic immune function in females. Moreover, we confirmed that PXR is a sensor of gut microbiota-derived signals and demonstrated that the microbiota–PXR interaction controls the host’s hepatic lipid and xenobiotic metabolism in a sexually dimorphic manner, with possible relevance to liver disease. Our results open a new metagenomic perspective on sexually dimorphic and interindividual differences in pharmacokinetics and sensitivity to environmental toxicity, and highlight a potential new mechanism for microbiota-targeted vs. host-targeted drug interaction.

## Supplementary Information

**Additional file 1 **Effect of ATB treatment. **(**A&C) Mean water intake per cage during ATB treatment in (A) males and (C) females. **(**B&D) Organ weights in (B) males and (D) females. (E&G) Fecal anaerobic colony counts in (E) males and (G) females. (F&H) Relative short chain fatty acids in caecal content from (F) males and (H) females. (I&J) Plasma biochemistry in (I) males and (J) females. Data represent mean ± SEM and were analyzed using Kruskal-Wallis test followed by Dunn’s multiple comparisons tests. AUC: Area Under the Curve; a.u.: arbitrary units; ALT: alanine-aminotransferases; HDL: high density lipoproteins; LDL: low density lipoproteins.

**Additional file 2.** Oligonucleotide sequences for real-time PCR.

**Additional file 3.** List of all genes detected in the microarray experiment, log(fold-changes), raw p-values and corrected p-values for inter-group comparisons.

**Additional file 4 **Caecal microbiota comparison of *Pxr*^*-/-*^ *vs. Pxr*^*+/+*^ male and female mice. (A) α-diversity at the OTU level in *Pxr*^*-/-*^ *vs. Pxr*^*+/+*^ males. (B) α-diversity at the OTU levels in *Pxr*^*-/-*^ *vs. Pxr*^*+/+*^ females. (C) PLS-DA at the OTU level. Each dot represents one individual sample. (D) Composition of the bacterial phyla in males. (E) Composition of the bacterial phyla in females. (F) Comparison of caecal microbiota was performed with LDA effect size (LEfSe). The red area indicates the over-abundance in *Pxr*^-/-^ male microbiota where the green area indicated the over-abundance in *Pxr*^+/+^ male microbiota. (G) Comparison of caecal microbiota was performed with LDA effect size (LEfSe). The red area indicates the over-abundance in *Pxr*^-/-^ female microbiota where the green area indicated the over-abundance in *Pxr*^+/+^ female microbiota. (H) Relative abundances of some bacterial genera identified as significantly different between groups using LEfSe analysis. * = Significantly different (p<0.05) according to Mann-Whitney test between *Pxr*^*-/-*^ *vs. Pxr*^*+/+*^ in each sex.

**Additional file 5 **Gene lists and GO enrichment analyses for *Pxr*^-/-^ *vs Pxr*^+/+^ transcriptome comparisons. (A) List of genes significantly up-regulated in males *Pxr*^-/-^ compared to males *Pxr*^+/+^. (B) GO term enrichment analysis using the list of genes significantly up-regulated in males *Pxr*^-/-^ compared to males *Pxr*^+/+^. Metabolic pathways (summary) were considered as significantly enriched when q-value < 0.05. (C) List of genes significantly up-regulated in females *Pxr*^-/-^ compared to females *Pxr*^+/+^. (D) GO term enrichment analysis using the list of genes significantly up-regulated in females *Pxr*^-/-^ compared to females *Pxr*^+/+^. Metabolic pathways (summary) were considered as significantly enriched when q-value < 0.05. (E) List of genes significantly down-regulated in males *Pxr*^-/-^ compared to males *Pxr*^+/+^. (F) GO term enrichment analysis using the list of genes significantly up-regulated in males *Pxr*^-/-^ compared to males *Pxr*^+/+^. No summary metabolic pathways (summary) were considered as significantly enriched with q-value < 0.05. (A) List of genes significantly down-regulated in females *Pxr*^-/-^ compared to females *Pxr*^+/+^. (B) GO term enrichment analysis using the list of genes significantly down-regulated in females *Pxr*^-/-^ compared to females *Pxr*^+/+^. Metabolic pathways (summary) were considered as significantly enriched when q-value < 0.05.

**Additional file 6 **Gene lists for sex-biased genes. (A) List of genes significantly higher in males *Pxr*^+/+^ compared to females *Pxr*^+/+^ (male-biased genes). (B) List of genes significantly higher in males *Pxr*^-/-^ compared to females *Pxr*^-/-^. (C) List of genes significantly higher in males *Pxr*^+/+^ ATB compared to females *Pxr*^+/+^ ATB. (D) List of genes significantly higher in females *Pxr*^+/+^ compared to males *Pxr*^+/+^ (female-biased genes). (E) List of genes significantly higher in females *Pxr*^-/-^ compared to males *Pxr*^-/-^. (F) List of genes significantly higher in females *Pxr*^+/+^ ATB compared to males *Pxr*^+/+^ ATB.

**Additional file 7 **Effect of PXR deletion on sexually-dimorphic gene expression. (A) Number of male-biased genes (established in *Pxr*^+/+^ mice, see methods) that remain male-biased in *Pxr*^-/-^ mice. (B) Number of female-biased genes (established in *Pxr*^+/+^ mice, see methods) that remain female-biased in *Pxr*^-/-^ mice. (C) Hierarchical clustering of all genes significantly different for at least one comparison between the 4 experimental groups (*Pxr*^+/+^ males, *Pxr*^+/+^ females, *Pxr*^-/-^ males, *Pxr*^-/-^ females. (D) Average expression Z-scores over gene clusters defined in (C). (E) Pathway enrichment analysis of the 1419 hepatic genes from cluster 1.

**Additional file 8 **Gene lists and GO enrichment analyses for ATB- vs. Cont. transcriptome comparisons. (A) List of genes significantly up-regulated in males *Pxr*^+/+^ ATB compared to males *Pxr*^+/+^ Cont. (B) GO term enrichment analysis using the list of genes significantly up-regulated in males *Pxr*^+/+^ ATB compared to males *Pxr*^+/+^ Cont. Metabolic pathways were considered as significantly enriched when adjusted P-value < 0.05. (C) List of genes significantly down-regulated in males *Pxr*^+/+^ ATB compared to males *Pxr*^+/+^ Cont. (D) GO term enrichment analysis using the list of genes significantly down-regulated in males *Pxr*^+/+^ ATB compared to males *Pxr*^+/+^ Cont. Metabolic pathways were considered as significantly enriched when adjusted P-value < 0.05. (E) List of genes significantly up-regulated in males *Pxr*^-/-^ ATB compared to males *Pxr*^-/-^ Cont. (F) List of genes significantly down-regulated in males *Pxr*^-/-^ ATB compared to males *Pxr*^-/-^ Cont. (G) List of genes significantly up-regulated in females *Pxr*^+/+^ ATB compared to females *Pxr*^+/+^ Cont. (H) List of genes significantly down-regulated in females *Pxr*^+/+^ ATB compared to females *Pxr*^+/+^ Cont. (I) List of genes significantly up-regulated in females *Pxr*^-/-^ ATB compared to females *Pxr*^-/-^ Cont. (J) GO term enrichment analysis using the list of genes significantly down-regulated in females *Pxr*^-/-^ ATB compared to males *Pxr*^-/-^ Cont. No metabolic pathways was found significantly enriched at adjusted P-value < 0.05. (K) List of genes significantly down-regulated in females *Pxr*^-/-^ ATB compared to females *Pxr*^-/-^ Cont.

**Additional file 9.** Lack of gut microbiota affects the hepatic transcriptome in a sexually dimorphic manner. Publically available RNA-sequencing data (GSE114400) comparing the hepatic transcriptome of GF and Conv male and female mice was re-analyzed. (A) Venn diagram representing the number of genes overexpressed in GF vs. Conv mice in males and females and the overlap between the 2 lists of genes. (B) Pathway enrichment analysis of the 587 hepatic genes overexpressed in GF vs. Conv males and not in GF vs Conv females. (C) Network plot of the enriched terms depicted in (B) colored by cluster ID, where nodes that share the same cluster ID are typically close to each other. Colors of the cluster ID are shown in (B). (D) Pathway enrichment analysis of the 200 hepatic genes overexpressed in GF vs. Conv females and not in GF vs Conv males. (E) Venn diagram representing the number of genes significantly underexpressed in GF vs. Conv mice in males and females and the overlap between the 2 lists of genes. (F) Pathway enrichment analysis of the 624 hepatic genes underexpressed in GF vs. Conv males and not in GF vs Conv females. (G) Network plot of the enriched terms depicted in (F) colored by cluster ID, where nodes that share the same cluster ID are typically close to each other. Colors of the cluster ID are shown in (F). (H) Pathway enrichment analysis of the 338 hepatic genes overexpressed in GF vs. Conv females and not in GF vs Conv males.

**Additional file 10 **Effect of ATB-treatment on sexually-dimorphic gene expression. (A) Number of male-biased genes (established in *Pxr*^+/+^ mice, see methods) that remain male-biased in ATB-treated *Pxr*^+/+^ mice. (B) Number of female-biased genes (established in *Pxr*^+/+^ mice, see methods) that remain female-biased in ATB-treated *Pxr*^+/+^ mice. (C) Hierarchical clustering of all genes significantly different for at least one comparison between the 4 experimental groups (*Pxr*^+/+^ males, *Pxr*^+/+^ females, *Pxr*^+/+^ ATB-treated males, *Pxr*^+/+^ ATB-treated females. (D) Average expression Z-scores over gene clusters defined in (C).

**Additional file 11 **Expression of Pxr, Cyp3a11 and Cyp2c55 mRNA in liver from *Pxr*^*+/+*^ Cont males and females analyzed using RT-qPCR.

**Additional file 12.** Effect of gut microbiota-PXR interaction on hepatic fatty acid and xenobiotic metabolism in female mice. (A) RT-qPCR analysis of hepatic genes involved in fatty-acid homeostasis. (B) Relative abundance of hepatic fatty acids. (C) Hepatic neutral lipid quantification. (D) RT-qPCR analysis of hepatic genes involved in xenobiotic metabolism.

**Additional file 13.** [^14^C]-testosterone metabolites structure hypotheses based on their mass and on the similarity of their retention time with authentic standards.

## Data Availability

The datasets supporting the conclusions of this article are available in the NCBI GEO repository under the accession numbers GSE31115, GSE71628, GSE114400, GSE123804 and GSE156837. The sequencing datasets were deposited in the European Nucleotide Archive under accession number PRJEB39629.

## Abbreviations

PXR: Pregnane X receptor
NAFLD: Nonalcoholic fatty liver disease
GF: Germ-free
Conv.: Conventional
ATB: Antibiotics
ZT: Zeitgeber time
PCN: Pregnenolone 16α-carbonitrile
ALT: Alanine transaminase
HDL–LDL: High- or low-density lipoprotein
FFA: Free fatty acids
GST: Glutathione S-transferase
OTU: Operational taxonomic unit
LDA: Linear discriminant analysis
LEfSe: Linear discriminant analysis effect size
CYP: Cytochrome P450
PLS-DA: Partial least square–discriminant analysis
