# Supplementary material for: The pregnane X receptor drives sexually dimorphic hepatic changes in lipid and xenobiotic metabolism in response to gut microbiota in mice
Source: Microbiome. 2021 Apr 20;9:93. doi: 10.1186/s40168-021-01050-9 (PMC8059225; doi:10.1186/s40168-021-01050-9)

Additional file 1: Effect of ATB treatment.

(A&C) Mean water intake per cage during ATB treatment in (A) males and (C) females.
(B&D) Organ weights in (B) males and (D) females.
(E&G) Fecal anaerobic colony counts in (E) males and (G) females.
(F&H) Relative short chain fatty acids in caecal content from (F) males and (H) females.
(I&J) Plasma biochemistry in (I) males and (J) females.
Data represent mean ± SEM and were analyzed using Kruskal-Wallis test followed by Dunn's multiple comparisons tests. AUC: Area Under the Curve; a.u.: arbitrary units; ALT: alanine-aminotransferases; HDL: high density lipoproteins; LDL: low density lipoproteins.

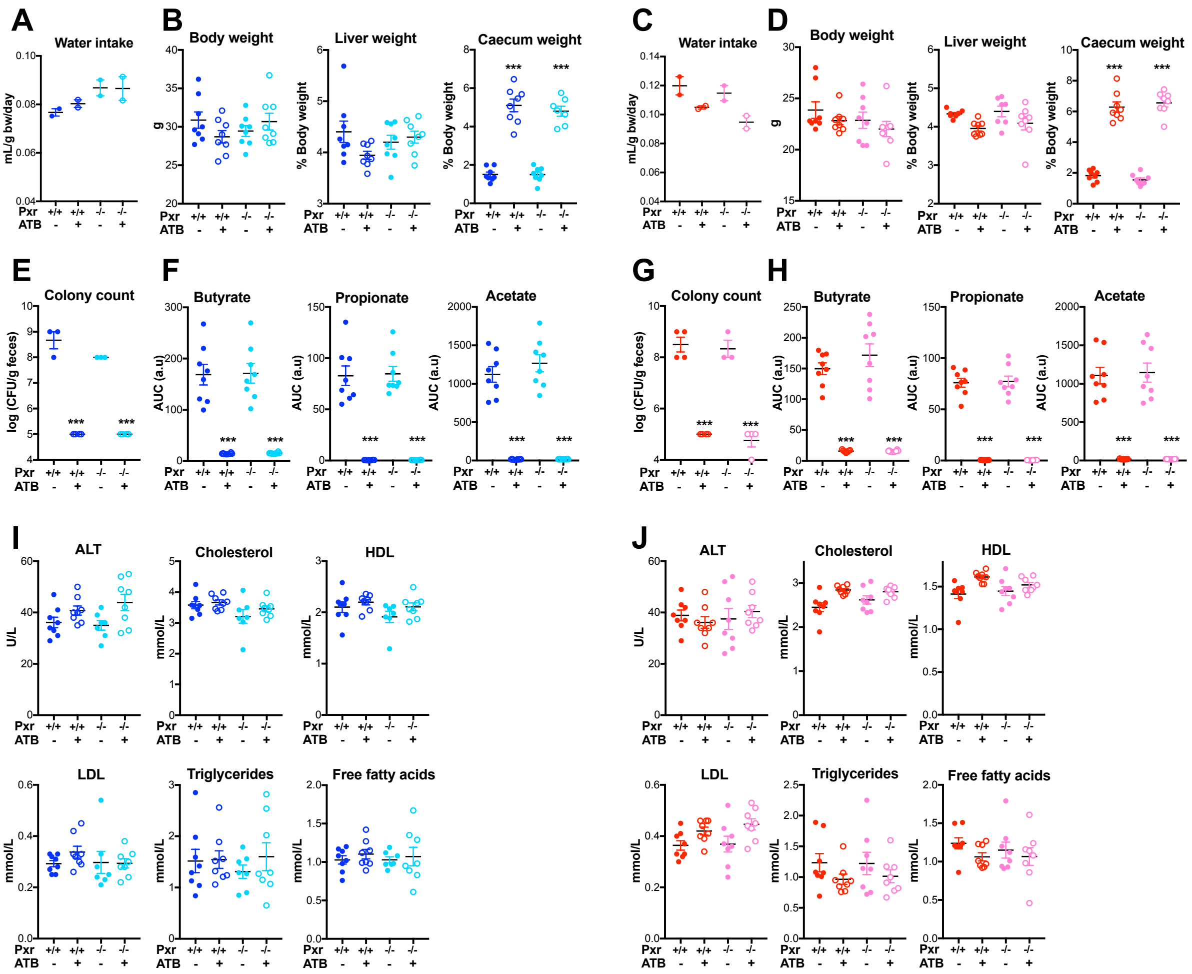

Supplement: Supplementary file 2 — Additional file 1 Effect of ATB treatment. (A&C) Mean water intake per cage during ATB treatment in (A) males and (C) females. (B&D) Organ weights in (B) males and (D) females. (E&G) Fecal anaerobic colony counts in (E) males and (G) females. (F&H) Relative short chain fatty acids in caecal content from (F) males and (H) females. (I&J) Plasma biochemistry in (I) males and (J) females. Data represent mean ± SEM and were analyzed using Kruskal-Wallis test followed by Dunn’s multiple comparisons tests. AUC: Area Under the Curve; a.u.: arbitrary units; ALT: alanine-aminotransferases; HDL: high density lipoproteins; LDL: low density lipoproteins. [file 40168_2021_1050_MOESM2_ESM.pdf]
