# Supplementary material for: The pregnane X receptor drives sexually dimorphic hepatic changes in lipid and xenobiotic metabolism in response to gut microbiota in mice
Source: Microbiome. 2021 Apr 20;9:93. doi: 10.1186/s40168-021-01050-9 (PMC8059225; doi:10.1186/s40168-021-01050-9)
Supplement: Supplementary file 3 — Additional file 2. Oligonucleotide sequences for real-time PCR. [file 40168_2021_1050_MOESM3_ESM.pdf]

Additional file 2. Oligonucleotide sequences for real-time PCR.

Gene	NCBI Refseq	Forward primer (5'-3')	Reverse primer (5'-3')
Cyp2b9	NM_010000	CTTTGCTGGAACTGAGACCACA	GATCTGAAAATCTCTGAATCTCATGG
Cyp2c55	AY206875	TTGTGGAAGAGCTAAGAAAAGCAAAT	GAGCACAGCTCAGGATGAATGT
Cyp3a11	NM_007818	TCACACACACAGTTGTAGGCAGAA	GTTTACGAGTCCCATATCGGTAGAG
Elovl2	NM_019423	GCAGAAGGAAGGCGGCTAC	CGCGAACTCGAGAATCTCGT
Elovl3	NM_007703	GCCTCTCATCCTCTGGTCCT	TGCCATAAACTTCCACATCCT
Elovl5	NM_134255	TCGATGCGTCACTCAGTACCTATT	ATTTTGGTCCCAGCCATACAAT
Elovl6	NM_130450	TCTGATGAACAAGCGAGCCA	TGGTCATCAGAATGTACAGCATGT
Fasn	NM_007988	AGTCAGCTATGAAGCAATTGTGGA	CACCCAGACGCCAGTGTTC
Fgf21	NM_020013	AAAGCCTCTAGGTTTCTTTGCCA	CCTCAGGATCAAAGTGAGGCG
