# Supplementary material for: The pregnane X receptor drives sexually dimorphic hepatic changes in lipid and xenobiotic metabolism in response to gut microbiota in mice
Source: Microbiome. 2021 Apr 20;9:93. doi: 10.1186/s40168-021-01050-9 (PMC8059225; doi:10.1186/s40168-021-01050-9)

Additional file 4: Caecal microbiota comparison of *Pxr*$^{-/-}$ *vs. Pxr*$^{+/+}$ male and female mice.
(A) α-diversity at the OTU level in *Pxr*$^{-/-}$ *vs. Pxr*$^{+/+}$ males.
(B) α-diversity at the OTU levels in *Pxr*$^{-/-}$ *vs. Pxr*$^{+/+}$ females.
(C) PLS-DA at the OTU level. Each dot represents one individual sample.
(D) Composition of the bacterial phyla in males.
(E) Composition of the bacterial phyla in females.
(F) Comparison of caecal microbiota was performed with LDA effect size (LEfSe). The red area indicates the over-abundance in *Pxr*$^{-/-}$ male microbiota where the green area indicated the over-abundance in *Pxr*$^{+/+}$ male microbiota.
(G) Comparison of caecal microbiota was performed with LDA effect size (LEfSe). The red area indicates the over-abundance in *Pxr*$^{-/-}$ female microbiota where the green area indicated the over-abundance in *Pxr*$^{+/+}$ female microbiota.
(H) Relative abundances of some bacterial genera identified as significantly different between groups using LEfSe analysis.
* = Significantly different ($p<0.05$) according to Mann-Whitney test between *Pxr*$^{-/-}$ *vs. Pxr*$^{+/+}$ in each sex.

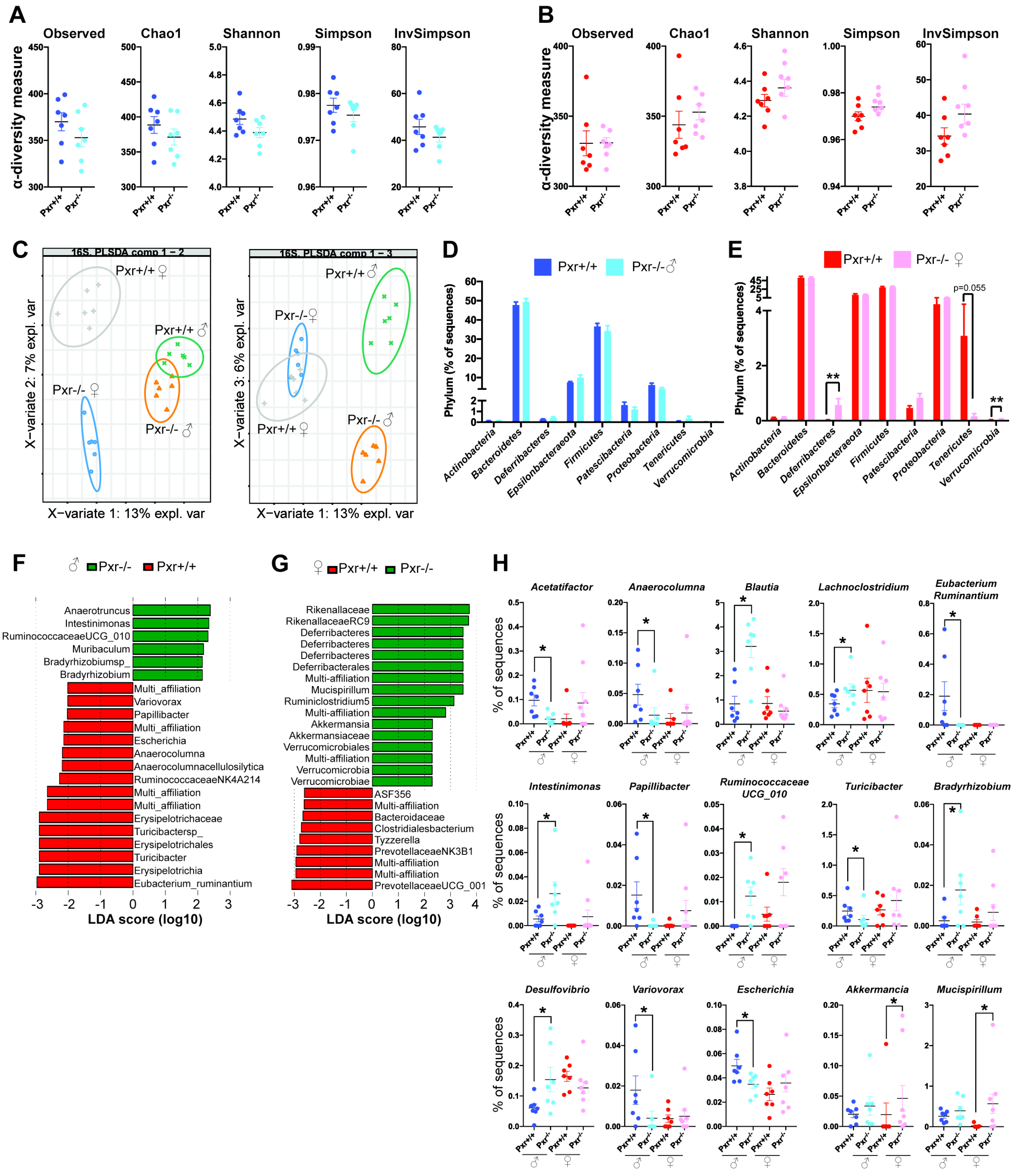

Supplement: Supplementary file 5 — Additional file 4 Caecal microbiota comparison of Pxr-/- vs. Pxr+/+ male and female mice. (A) α-diversity at the OTU level in Pxr-/- vs. Pxr+/+ males. (B) α-diversity at the OTU levels in Pxr-/- vs. Pxr+/+ females. (C) PLS-DA at the OTU level. Each dot represents one individual sample. (D) Composition of the bacterial phyla in males. (E) Composition of the bacterial phyla in females. (F) Comparison of caecal microbiota was performed with LDA effect size (LEfSe). The red area indicates the over-abundance in Pxr-/- male microbiota where the green area indicated the over-abundance in Pxr+/+ male microbiota. (G) Comparison of caecal microbiota was performed with LDA effect size (LEfSe). The red area indicates the over-abundance in Pxr-/- female microbiota where the green area indicated the over-abundance in Pxr+/+ female microbiota. (H) Relative abundances of some bacterial genera identified as significantly different between groups using LEfSe analysis. * = Significantly different (p<0.05) according to Mann-Whitney test between Pxr-/- vs. Pxr+/+ in each sex. [file 40168_2021_1050_MOESM5_ESM.pdf]
