# Supplementary material for: The pregnane X receptor drives sexually dimorphic hepatic changes in lipid and xenobiotic metabolism in response to gut microbiota in mice
Source: Microbiome. 2021 Apr 20;9:93. doi: 10.1186/s40168-021-01050-9 (PMC8059225; doi:10.1186/s40168-021-01050-9)

Additional file 9: Lack of gut microbiota affects the hepatic transcriptome in a sexually dimorphic manner.

Publically available RNA-sequencing data (GSE114400) comparing the hepatic transcriptome of GF and Conv male and female mice was re-analyzed.

(A) Venn diagram representing the number of genes overexpressed in GF vs. Conv mice in males and females and the overlap between the 2 lists of genes.

(B) Pathway enrichment analysis of the 587 hepatic genes overexpressed in GF vs. Conv males and not in GF vs Conv females.

(C) Network plot of the enriched terms depicted in (B) colored by cluster ID, where nodes that share the same cluster ID are typically close to each other. Colors of the cluster ID are shown in (B).

(D) Pathway enrichment analysis of the 200 hepatic genes overexpressed in GF vs. Conv females and not in GF vs Conv males.

(E) Venn diagram representing the number of genes significantly underexpressed in GF vs. Conv mice in males and females and the overlap between the 2 lists of genes.

(F) Pathway enrichment analysis of the 624 hepatic genes underexpressed in GF vs. Conv males and not in GF vs Conv females.

(G) Network plot of the enriched terms depicted in (F) colored by cluster ID, where nodes that share the same cluster ID are typically close to each other. Colors of the cluster ID are shown in (F).

(H) Pathway enrichment analysis of the 338 hepatic genes overexpressed in GF vs. Conv females and not in GF vs Conv males.

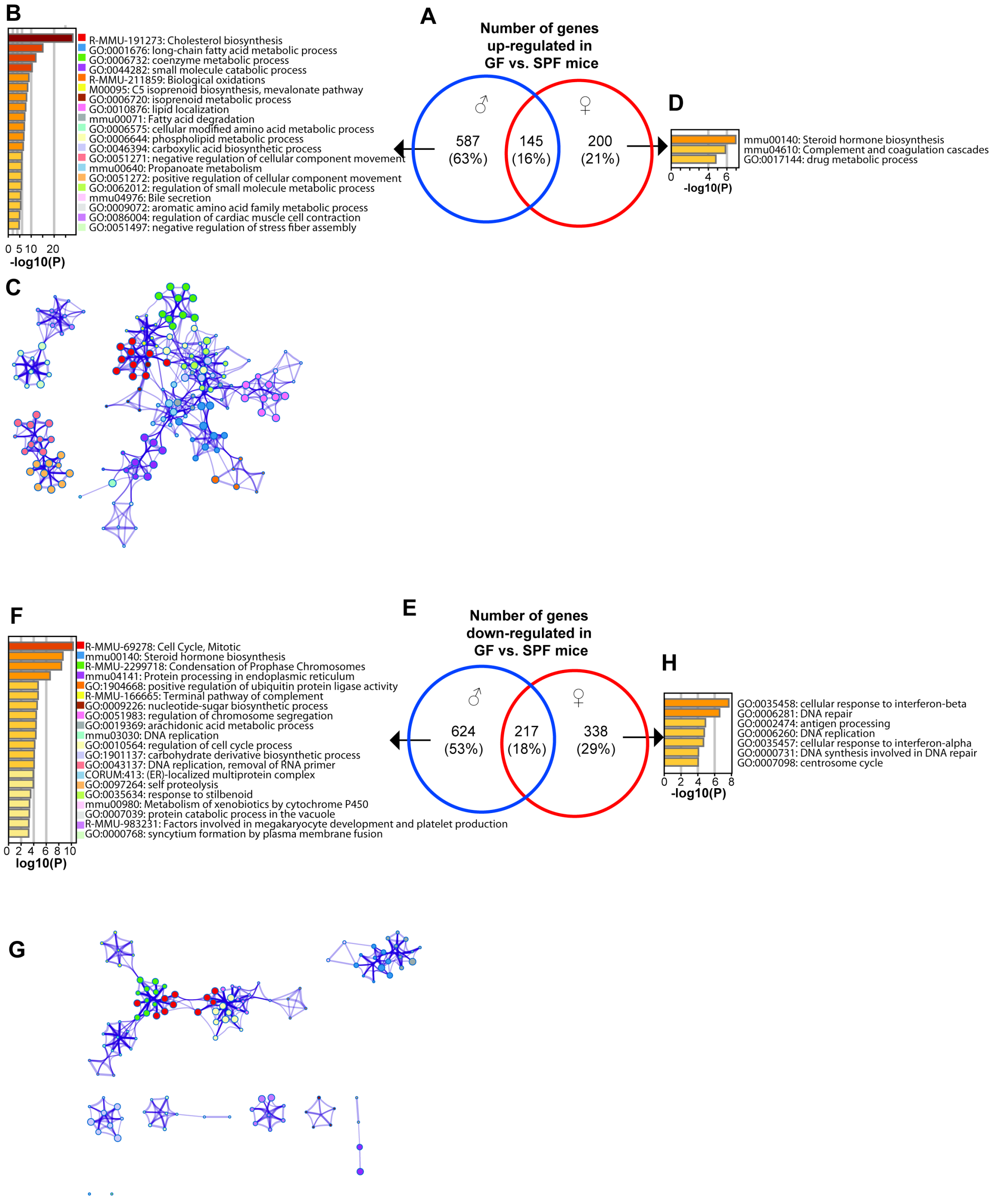

Supplement: Supplementary file 10 — Additional file 9. Lack of gut microbiota affects the hepatic transcriptome in a sexually dimorphic manner. Publically available RNA-sequencing data (GSE114400) comparing the hepatic transcriptome of GF and Conv male and female mice was re-analyzed. (A) Venn diagram representing the number of genes overexpressed in GF vs. Conv mice in males and females and the overlap between the 2 lists of genes. (B) Pathway enrichment analysis of the 587 hepatic genes overexpressed in GF vs. Conv males and not in GF vs Conv females. (C) Network plot of the enriched terms depicted in (B) colored by cluster ID, where nodes that share the same cluster ID are typically close to each other. Colors of the cluster ID are shown in (B). (D) Pathway enrichment analysis of the 200 hepatic genes overexpressed in GF vs. Conv females and not in GF vs Conv males. (E) Venn diagram representing the number of genes significantly underexpressed in GF vs. Conv mice in males and females and the overlap between the 2 lists of genes. (F) Pathway enrichment analysis of the 624 hepatic genes underexpressed in GF vs. Conv males and not in GF vs Conv females. (G) Network plot of the enriched terms depicted in (F) colored by cluster ID, where nodes that share the same cluster ID are typically close to each other. Colors of the cluster ID are shown in (F). (H) Pathway enrichment analysis of the 338 hepatic genes overexpressed in GF vs. Conv females and not in GF vs Conv males. [file 40168_2021_1050_MOESM10_ESM.pdf]
