# Supplementary material for: The pregnane X receptor drives sexually dimorphic hepatic changes in lipid and xenobiotic metabolism in response to gut microbiota in mice
Source: Microbiome. 2021 Apr 20;9:93. doi: 10.1186/s40168-021-01050-9 (PMC8059225; doi:10.1186/s40168-021-01050-9)

Additional file 10: Effect of ATB-treatment on sexually-dimorphic gene expression

(A) Number of male-biased genes (established in *Pxr*$^{+/+}$ mice, see methods) that remain male-biased in ATB-treated *Pxr*$^{+/+}$ mice.

(B) Number of female-biased genes (established in *Pxr*$^{+/+}$ mice, see methods) that remain female-biased in ATB-treated *Pxr*$^{+/+}$ mice.

(C) Hierarchical clustering of all genes significantly different for at least one comparison between the 4 experimental groups (*Pxr*$^{+/+}$ males, *Pxr*$^{+/+}$ females, *Pxr*$^{+/+}$ ATB-treated males, *Pxr*$^{+/+}$ ATB-treated females.

(D) Average expression Z-scores over gene clusters defined in (C).

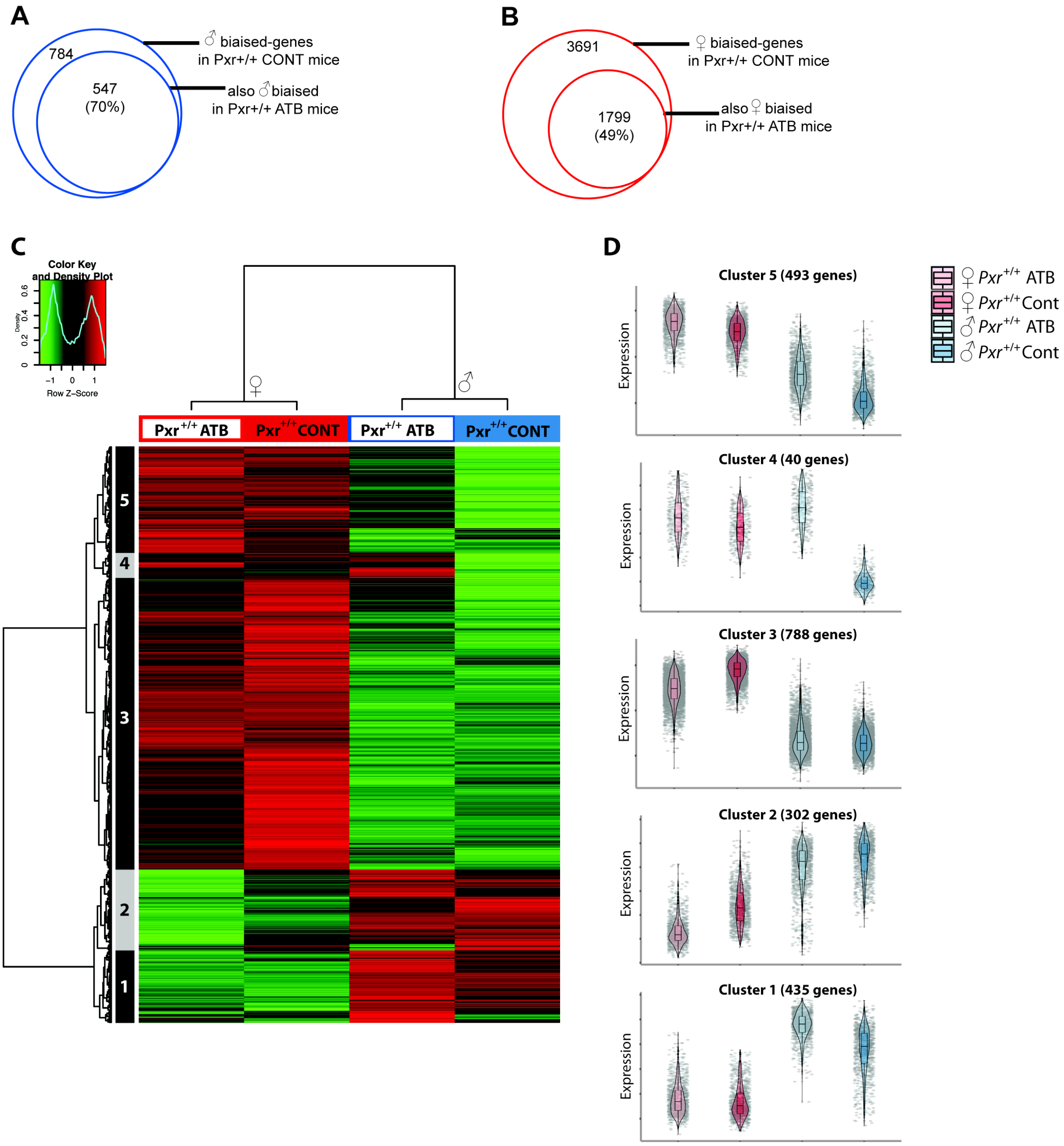

Supplement: Supplementary file 11 — Additional file 10 Effect of ATB-treatment on sexually-dimorphic gene expression. (A) Number of male-biased genes (established in Pxr+/+ mice, see methods) that remain male-biased in ATB-treated Pxr+/+ mice. (B) Number of female-biased genes (established in Pxr+/+ mice, see methods) that remain female-biased in ATB-treated Pxr+/+ mice. (C) Hierarchical clustering of all genes significantly different for at least one comparison between the 4 experimental groups (Pxr+/+ males, Pxr+/+ females, Pxr+/+ ATB-treated males, Pxr+/+ ATB-treated females. (D) Average expression Z-scores over gene clusters defined in (C). [file 40168_2021_1050_MOESM11_ESM.pdf]
