# Supplementary material for: The pregnane X receptor drives sexually dimorphic hepatic changes in lipid and xenobiotic metabolism in response to gut microbiota in mice
Source: Microbiome. 2021 Apr 20;9:93. doi: 10.1186/s40168-021-01050-9 (PMC8059225; doi:10.1186/s40168-021-01050-9)

Additional file 11: Expression of Pxr, Cyp3a11 and Cyp2c55 mRNA in liver from *Pxr*$^{+/+}$ Cont males and females analyzed using RT-qPCR.

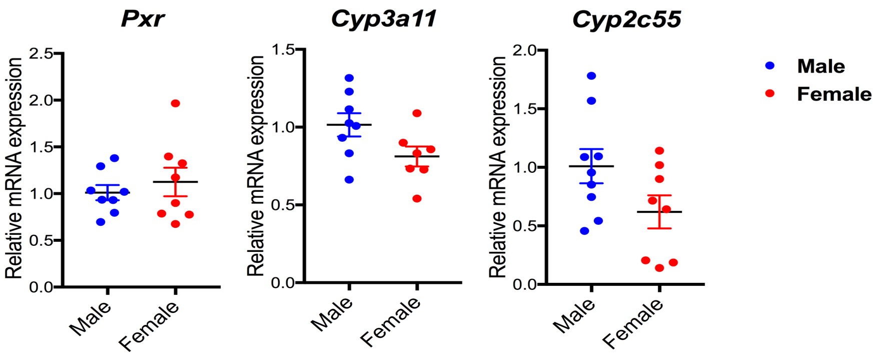

Supplement: Supplementary file 12 — Additional file 11 Expression of Pxr, Cyp3a11 and Cyp2c55 mRNA in liver from Pxr+/+ Cont males and females analyzed using RT-qPCR. [file 40168_2021_1050_MOESM12_ESM.pdf]
