# Supplementary material for: The pregnane X receptor drives sexually dimorphic hepatic changes in lipid and xenobiotic metabolism in response to gut microbiota in mice
Source: Microbiome. 2021 Apr 20;9:93. doi: 10.1186/s40168-021-01050-9 (PMC8059225; doi:10.1186/s40168-021-01050-9)

Additional file 12: Effect of gut microbiota-PXR interaction on hepatic fatty acid and xenobiotic metabolism in female mice

(A) RT-qPCR analysis of hepatic genes involved in fatty-acid homeostasis.
(B) Relative abundance of hepatic fatty acids.
(C) Hepatic neutral lipid quantification.
(D) RT-qPCR analysis of hepatic genes involved in xenobiotic metabolism.

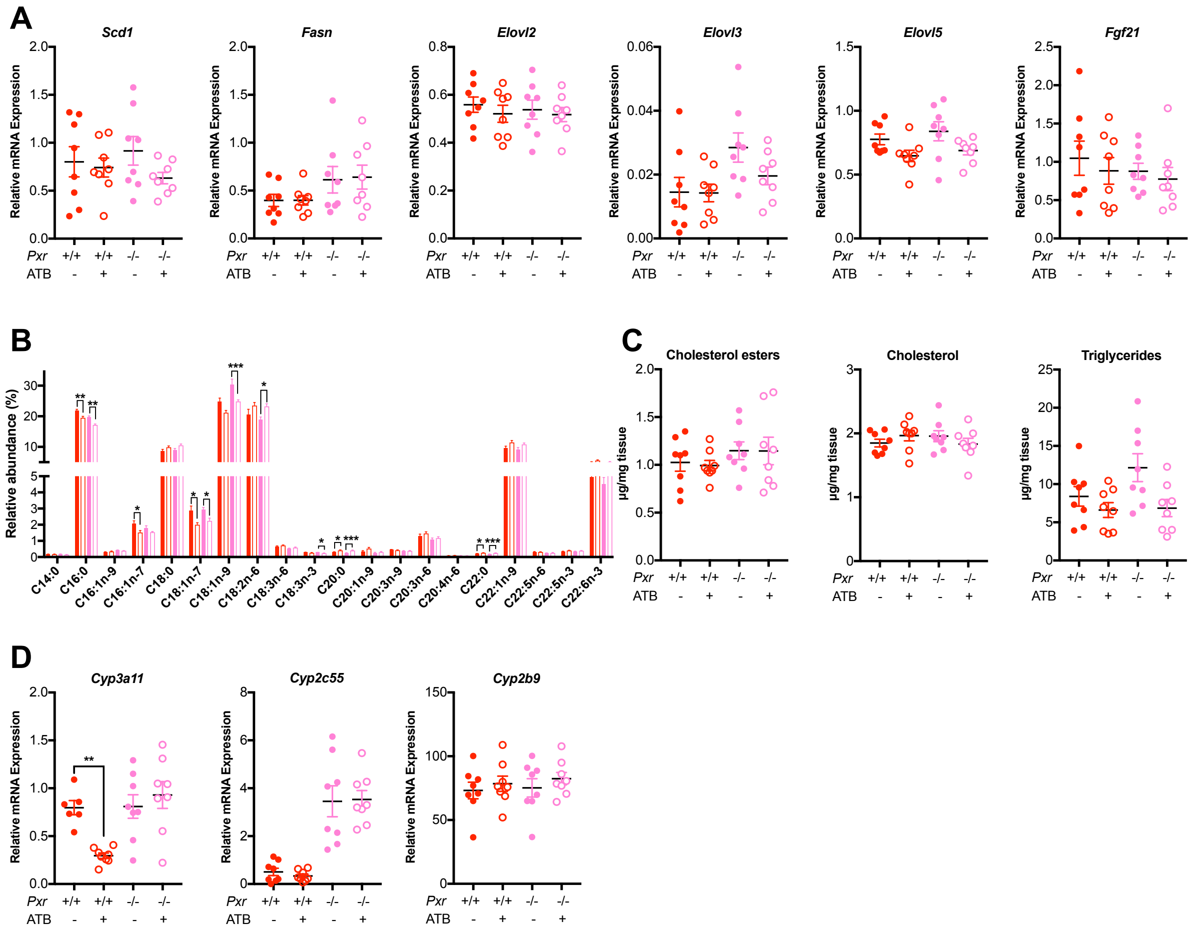

Supplement: Supplementary file 13 — Additional file 12. Effect of gut microbiota-PXR interaction on hepatic fatty acid and xenobiotic metabolism in female mice. (A) RT-qPCR analysis of hepatic genes involved in fatty-acid homeostasis. (B) Relative abundance of hepatic fatty acids. (C) Hepatic neutral lipid quantification. (D) RT-qPCR analysis of hepatic genes involved in xenobiotic metabolism. [file 40168_2021_1050_MOESM13_ESM.pdf]
