# Supplementary material for: The pregnane X receptor drives sexually dimorphic hepatic changes in lipid and xenobiotic metabolism in response to gut microbiota in mice
Source: Microbiome. 2021 Apr 20;9:93. doi: 10.1186/s40168-021-01050-9 (PMC8059225; doi:10.1186/s40168-021-01050-9)
Supplement: Supplementary file 14 — Additional file 13. [14C]-testosterone metabolites structure hypotheses based on their mass and on the similarity of their retention time with authentic standards. [file 40168_2021_1050_MOESM14_ESM.pdf]

Additional file 13: [^{14}C]-testosterone metabolites structure hypotheses based on their mass and on the similarity of their retention time with authentic standards

LC-MS Retention time (min)	Corresponding radio-HPLC Retention time (min)	Metabolites		m/z
7.22	7.9	hydroxytestosterone	OH-Testo	305.2111
7.71			OH-Δ4-dione	303.1955
8.2	8.8	15β-hydroxytestosterone	15β-OH-Testo	305.2111
8.25		6α-hydroxytestosterone	6α-OH-Testo	305.2111
8.27			Δ-Testo	287.2006
8.44			OH-Δ4-dione	303.1955
8.9		hydroxytestosterone	OH-Testo	305.2111
9.2	9.7	7α-hydroxytestosterone	7α-OH-Testo	305.2111
9.21			Δ-Testo	287.2006
9.66	10.2	6β-hydroxytestosterone	6β-OH-Testo	305.2111
10.18			OH-Δ4-dione	303.1955
11.59	12.2	16α-hydroxytestosterone	16α-OH-Testo	305.2111
12.65	13.5	11α-hydroxytestosterone	11α-OH-Testo	305.2111
12.67			Δ-Testo	287.2006
13.0			OH-Δ4-dione	303.1955
13.72	14.4		OH-Δ4-dione	303.1955
15.42	16.0	16β-hydroxytestosterone	16β-OH-Testo	305.2111
16.15		4-Androsten-16α-ol-3,17-dione	16α-OH-Δ4-dione	303.1955
25.7	26.3	4,6-androstadien-17β-ol-3-one	Δ6-Testo	287.2006
28.63	29.8	Testosterone	Testo	289.2162
32.34	33	Androstenedione = 4-androsten-3,17-dione	Δ4-Testo	287.2006
